# Human and Chimp: Can Our Genes Tell the Story of Our Divergence?

**DOI:** 10.1371/journal.pbio.0030202

**Published:** 2005-05-03

Since humans and chimpanzees forged separate evolutionary paths some 5 million to 6 million years ago, we shed our hirsute coat and heavy brow, mastered bipedal locomotion, and acquired a knack for abstract thought while our next of kin learned to use tools, created a complex communication system, and developed the skills to construct tree-bound nests high above the forest floor. We differ by just a tad over 1% at the DNA sequence level, yet scientists predict that both species should harbor genetic footprints of our divergence.

One way to find such genetic signatures is to search for genes that reveal signs of natural selection. The assumption is that genes or genomic elements touched by natural selection will show more functionally significant molecular changes than unaffected regions. A 2003 study by Andrew Clarke et al. used this approach to identify human genes affected by positive selection—that is, selection that preserves new genetic variants—by comparing 7,645 genes from humans to their chimp and mouse equivalents. Clarke et al. identified genes in several functional categories, including olfaction and hearing, and showed that positively selected genes are more likely to contain variations (called single nucleotide polymorphisms, or SNPs) associated with genetic diseases.

In a new study, Rasmus Nielsen, Michele Cargill, and their colleagues (many of whom participated in the 2003 study) compared 13,731 known genes in humans to their equivalents in chimps to find positively selected genes in both species. Nielsen et al. also identified many genes involved in sensory perception, as well as spermatogenesis, but found the strongest evidence for positive selection in genes related to immune defense. Immunity genes, the authors explain, were likely targeted throughout mammalian evolution, while the perception and olfactory genes probably reflect primate-specific adaptations. They also found a “surprising number” of tumor-suppression and apoptosis genes.[Fig pbio-0030202-g001]

**Figure pbio-0030202-g001:**
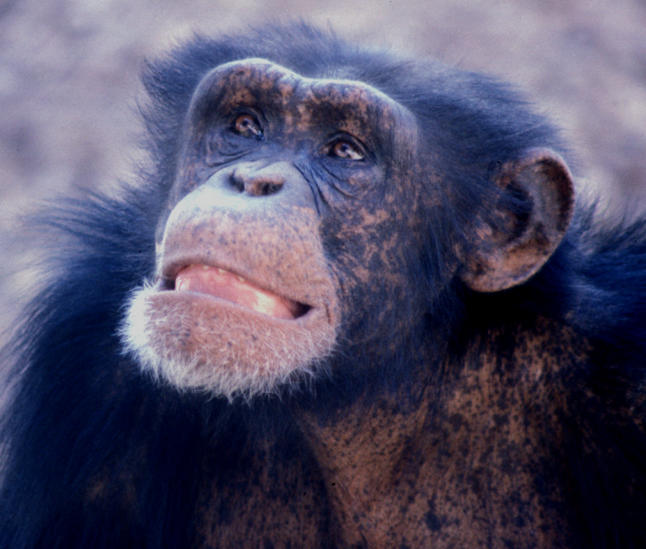
Young adult male chimpanzee (Photo: Frans de Waal, Emory University)

Mutations in coding DNA can be broadly classified into two groups: a nucleotide change can cause an amino acid substitution that either alters the encoded protein (called nonsynonymous mutation) or has no effect (synonymous mutation). Nielsen et al. used a statistical method that denotes positive selection based on the ratio of nonsynonymous to synonymous mutations. Thousands of genes failed to unambiguously pass this test, leaving 8,079 for further study; those that passed were grouped into functional categories, revealing the genes related to immunity, sensory perception, and spermatogenesis. Of genes associated with specific tissues, only testis-specific genes showed evidence of positive selection. Genes expressed in the brain appear to be under selective constraints, indicating that the genetic roots of our cognitive distance from chimps lies elsewhere, perhaps in how genes are regulated or organized.

The authors followed up the chimp–human comparison by analyzing the top 50 genes from their list in a group of African- and European-Americans. The data provide further support for the conclusion that these genes have undergone positive selection.

Nielsen et al. were surprised to find so many genes involved in tumor development and control among the top 50 positively selected genes (in both primates). The factors behind this pattern are unclear, but the authors suggest that studying the genes' other functions, in immunity or spermatogenesis, may offer clues to selective pressures—and it also raises some intriguing paradoxes. It could be, for example, that the overrepresentation of genes involved in tumor suppression, spermatogenesis, and apoptosis sets up competing interests on two fronts. Apoptosis normally eliminates up to 75% of sperm cells during spermatogenesis; mutations that protect sperm cells from apoptosis may be selected for, benefiting the cell but compromising the fitness of the organism. Positive selection for apoptosis avoidance in the germ line could subsequently increase the probability of cancer in body cells—apoptosis routinely eliminates unhealthy cells—pitting the “selfish interests of a germ cell” against the organism's interest in avoiding cancer.

Future studies will have to determine whether these explanations—of an evolutionary arms race—prove plausible. We're a long way from understanding why we're so different from our closest primate cousins, but this study provides plenty of tools, and hypotheses, to mine the tiny differences in our DNA for more clues.

